# Decline in RNA integrity of dry-stored soybean seeds correlates with loss of germination potential

**DOI:** 10.1093/jxb/erx100

**Published:** 2017-04-12

**Authors:** Margaret B Fleming, Christopher M Richards, Christina Walters

**Affiliations:** USDA-ARS, National Laboratory for Genetic Resource Preservation, Fort Collins, CO, USA

**Keywords:** Age, degradation, germination tests, RIN, RNA, seed longevity, seed storage, soybean, viability

## Abstract

This study investigates the relationship between germination ability and damage to RNA in soybean seeds (cv ‘Williams 82’) stored dry at 5 °C for 1–27 years. Total germination of 14 age cohorts harvested between 2015 and 1989 ranged from 100% to 3%. Germination decline followed classic seed viability kinetics, with symptomatic seed aging beginning after 17 years of storage. RNA integrity was assessed in dry seeds by electrophoresis of total RNA, followed by calculation of the RNA integrity number (RIN, Agilent Bioanalyzer software), which evaluates RNA fragment size distributions. Analysis of RNA extracted from cotyledons, embryonic axes, plumules, and seed coats across the range of age cohorts showed consistent RNA degradation: older seeds had over-representation of small RNAs compared with younger seeds, which had nearly a 2:1 ratio of 25S and 18S rRNAs. RIN values for cotyledons and embryonic axes from the same seed were correlated. Decline in RIN tracked reduced germination, with a pronounced decrease in RIN after 17 years of storage. This led to a high correlation between the mean RIN of cotyledon RNA and the total germination percentage (*R*^2^=0.91, *P*<0.0001). Despite this relationship, germinable and non-germinable seeds within cohorts could not be distinguished unless the RIN was <3.5, indicating substantial deterioration. Our work demonstrates that seed RNA incurs damage over time, observable in fragment size distributions. Under the experimental conditions used here, RIN appears to be a promising surrogate for germination tests used to monitor viability of stored seeds.

## Teaser

Deterioration of RNA in dry-stored seeds occurs slowly and contemporaneously with loss of viability, making RNA quality an excellent marker for changes in seed quality during long-term storage.

## Introduction

Seed longevity is regulated by a combination of interacting genetic and environmental factors, making the phenotype difficult to measure or predict. In addition to multilocus genetic inheritance (e.g. Clerkx *et al.*, 2004; Rajjou *et al.*, 2012; Agacka-Mołdoch *et al.*, 2016), several other factors influence how long seeds survive, including initial properties of the seed lot, parental conditions during seed growth and maturation, and seed storage conditions (Nagel and Borner, 2010; Walters *et al.*, 2010; He *et al.*, 2014; Agacka-Mołdoch *et al.*, 2016). The seed longevity phenotype is also difficult to assess because an unknown amount of time passes before detectable changes occur. In a homogeneous seed population, there is usually a threshold after which individuals die rapidly (Walters *et al.*, 2010, and references therein). Presumably, the threshold indicates accumulation of a critical level of damage to components essential for seed survival and germination. The process by which damage accumulates is termed ‘aging’. At present, no method reliably predicts when the aging threshold will occur in a particular seed lot, and therefore assessments of health in stored seeds rely on multiple germination assays over an extended period of time (FAO, 2014).

Currently, the most recognized symptoms of seed aging are the increasing proportion of individuals in the population (seed lot) that fail to germinate or that germinate slowly as storage time progresses. Yet, something must have happened to make the previously germinable seed unable to germinate (e.g. Roberts *et al.*, 1973; Walters *et al.*, 2010; Kranner *et al.*, 2011; Chen *et al.*, 2013; Sano *et al.*, 2016; Waterworth *et al.*, 2016, and references therein)—‘something’ that should be identifiable, measurable, and accumulate incrementally, providing detectable aging symptoms before reaching the aging threshold. Several researchers have suggested that RNA quality is the ‘weak link’ in the ability of aging seeds to germinate normally (e.g. Bray and Chow 1976; Brocklehurst and Fraser, 1980; Kranner *et al.*, 2011; Chen *et al.*, 2013). The connection between RNA and aging was suggested, in part, because protein synthesis is required and highly regulated during germination but *de novo* transcription is not (Rajjou *et al.*, 2004, 2012, and references therein; Bai *et al.*, 2017). Previous work shows a relationship between seed germination and rRNA banding patterns (Roberts *et al.*, 1973; Bray and Chow, 1976; Brocklehurst and Fraser, 1980; Thompson *et al.*, 1987; Kranner *et al.*, 2011; Chen *et al.*, 2013). Bray and Chow (1976) indicated more RNA fragmentation in dead seeds, compared with unaged, living counterparts. Chen *et al.* (2013) showed preliminary evidence of RNA degradation in seeds that deteriorated due to high moisture exposure. Viability and RNA integrity have also been linked in other anhydrobiotic organisms (Segev *et al.*, 2012).

It seems reasonable that RNA integrity and seed quality would be linked. RNA is a labile molecule because the 2' OH group, absent in DNA, readily hydrolyzes (Eigner *et al.*, 1961; Adams *et al.*, 1992). RNA is also single-stranded, making it vulnerable to degradation by reactive oxygen species (Bazin *et al.*, 2011). The innate low stability of RNA molecules and the need for a dynamic mRNA pool might explain why metabolically active cells tend to turn over defective RNA molecules rather than repair them (Dai *et al.*, 2016). An individual mRNA usually persists for only a few hours in hydrated systems. In contrast, limited metabolism in dry seeds precludes repair or turnover of damaged RNA (or any molecule) (Buitink and Leprince, 2008; Ogé *et al.*, 2008; Fernández-Marín *et al.*, 2013). Hence, damaged RNA may accumulate with storage time and eventually prohibit expression of gene products required for germination, both during and after translation; loss of translational capacity has been shown *in vivo* for low-viability seeds (Roberts *et al.*, 1973; Honda *et al.*, 2005; Wurtmann and Wolin, 2009; Graille and Séraphin, 2012; Simms *et al.*, 2014; Waterworth *et al.*, 2016). No doubt the cell suffers when damaged RNA is not processed and removed.

The process and specificity of RNA degradation are also major interests in the literature on biological collections because biorepositories seek to quantify the effects of pre-analytical treatments on subsequent utility of stored samples, especially those involved with human health (International Society for Biological and Environmental Repositories, 2012; Pazzagli *et al.*, 2013; Malentacchi *et al.*, 2014). Requirements for samples having high-quality RNA have led to more stringent storage procedures and shorter ‘expiration dates’ compared with studies using DNA or proteins (Shabihkhani *et al.*, 2014). The possibility that some mRNAs degrade before others during sample storage, or even after recovery from long-term storage, suggests that bias may be introduced during transcriptome profiling of samples retrieved from biorepositories (Sidova *et al.*, 2015; Wei *et al.*, 2016). Therefore, an overarching question is whether RNA degradation influences gene expression patterns and, ultimately, controls developmental switches. In seeds and spores, the switch from dormant to germinable has been shown to depend in part on targeted degradation of mRNA species involved in repression of germination (Johnson and Dyer, 2000; Bazin *et al.*, 2011; Segev *et al.*, 2012).

This study offers a unique opportunity to evaluate RNA stability within stored seeds and relate changes in RNA quality to physiological capacity for germination. Soybean seeds were stored under the same conditions for nearly 30 years, and seeds from some older harvests, or ‘age cohorts’, died during that period. Our goal was to describe the relationship between RNA integrity and germination potential among age cohorts. We used the RNA integrity number (RIN) as an accepted, standardized method to quantify RNA integrity (Schroeder *et al.*, 2006) and measured RIN as a function of tissue type and storage time. We tested the hypotheses that (i) RNA degraded within the 30 year storage period; (ii) initial quality and degradation rate were similar among different tissues, and (iii) RNA fragmentation patterns distinguished between seeds that were dead or alive. We believe these tests will contribute to better biological markers of seed aging as well as serve as a foundation for future studies examining changes in mRNA abundance associated with lost germination potential.

## Materials and methods

### Plant material

Test material was soybean (*Glycine max*, cv ‘Williams 82’) seeds grown in the US midwest between 1989 and 2015. Seeds were purchased or donated from various commercial or public sources: Illinois Foundation (1989–1996), United States Department of Agriculture soybean germplasm collection O8U-I-SI (1996–2008), and Missouri Foundation (2009–2015). Seeds were stored at 5 °C and ~35% relative humidity (RH) until use.

### Germination assay and calculation of longevity

Germination of 14 different age cohorts (1–27 years) was measured in 2016. Seeds were germinated in rolls of moist germination paper (Anchor, St. Paul, MN, USA) incubated in the dark using a 16/8 h temperature cycle of 25 °C/15 °C. Each germination assay comprised three rolls of 20 seeds each for a total of 60 seeds. Six days after sowing, seeds were scored as apparently healthy (radicle >5 mm), stunted (radicle emerging from the seed coat but <5 mm), or not germinated (no radicle emergence). Total germination for a seed lot was calculated by pooling counts of apparently healthy and stunted seed. Radicle length was also measured to indicate growth rate. Germination data (total and apparently healthy) were treated as proportion data (Crawley, 2007), and the response to storage time was modeled using the glm function with a binomial distribution available in the statistical package R (R Core Team, 2016). Time for germination percentage to decrease to 80, 50, and 10% (P80, P50, and P10, respectively) was calculated using the dose.p function available in R (R Core Team, 2016). We used the value of P80 to indicate the duration of the initial asymptomatic phase of seed deterioration (Mira *et al.*, 2016).

### RNA extraction

RNA was characterized from 10 of the 14 age cohorts, chosen to encompass the measured range of total germination (from 3% to 100%). RNA was extracted from dry embryonic axes, plumules, cotyledons, and seed coats from 2–10 individual seeds from each cohort (embryonic axis and cotyledon from four or five seeds; plumule from five other seeds; and seed coat from two seeds also used for plumule extraction); each tissue and seed was analyzed separately. Plumule tissue included no more than 1 mm of the axis below the cotyledonary node. Approximately 30 mg of cotyledon was sampled either from the portion directly subtending the embryonic axis (the axis was also sampled for RNA characterization) or from the portion distal to the embryonic axis. When the distal portion of the cotyledon was sampled, the remaining axis and cotyledon were tested for germination. In this way, we identified cotyledons from seeds that were and were not germinable. Hundreds of cotyledons were sampled in age cohorts with high germination (i.e. the 2- and 16-year-old seed lots).

Embryonic axis and cotyledon samples were ground in microcentrifuge tubes with a micropestle under liquid nitrogen in the presence of 1–2 mg of polyvinylpyrrolidone-40 (PVP-40). Plumule and seed coat samples were ground for 1 min using a steel shot #40 BB and a Retsch (Haan, Germany) Bead Mill set at 30 oscillations s^–1^, under liquid nitrogen and in the presence of PVP-40, added at a mass comparable with the seed sample (i.e. ~1 mg).

RNA was extracted from ground embryonic axis and cotyledon tissues using a modification of the MMY method (Mornkham *et al.*, 2013), described below. RNA was extracted from plumule and seed coat tissues using the Qiagen (Hilden, Germany) Plant RNEasy kit according to the manufacturer’s protocol. The final wash with 500 µl of buffer RPE was repeated to reduce carryover of guanidine hydrochloride. The Qiagen method yielded higher amounts of RNA in parallel extractions of cotyledon tissue, and both extraction methods gave similar electrophoretic patterns and RIN values, except that the retention of small RNAs was reduced with the column-based Qiagen method compared with the precipitation-based MMY method (data not shown).

For the modified MMY extraction method (Mornkham *et al.*, 2013), all solutions were made in diethylpyrocarbonate (DEPC)-treated water, and all incubations and centrifugations took place at room temperature (22 °C) unless stated otherwise. Chemicals were of molecular biology grade. Extraction buffer [0.9 ml of 8 M LiCl, 2% (w/v) PVP-40, 5% 2-mercaptoethanol] was added to ground samples, followed immediately by 350 µl of 100% ethanol. The samples were vortexed vigorously and incubated for 5 min before adding 100 µl of chloroform and gently mixing. The samples were centrifuged at 2000 *g* for 3 min. The upper aqueous phase was removed by pipetting, and 550 µl of chloroform and 550 µl of solubilization buffer [1.4% (w/v) SDS, 75 mM NaCl, 25 mM EDTA, 2% 2-mercaptoethanol] were added. The samples were again mixed gently, then centrifuged at 2000 *g* for 3 min. The supernatant was transferred to a new tube and 500 µl of TRIzol^®^ Reagent (Ambion, Carlsbad, CA, USA) and 100 µl of chloroform were added. After vortexing, samples were incubated for 2 min. They were then centrifuged at 16 000 *g* for 10 min. The aqueous phase was transferred to a new tube, and an equal volume of chloroform was added. After mixing, samples were incubated on a nutator for 10 min and then centrifuged at 16 000 *g* for 10 min. The chloroform extraction of the aqueous phase was repeated once. After the second chloroform extraction, the aqueous phase was transferred to a new tube and an equal volume of isopropanol was added. After mixing, RNA was precipitated by placing the samples at −20 °C for 15 min, then centrifuging them at 20 000 *g* for 10 min at 4 °C. The supernatant was decanted and the pellet was washed twice with 70% ethanol. The pellet was dried and resuspended in 20 µl of water. Samples were processed in batches of 11 and included an embryonic axis and cotyledon (same seed) from five age cohorts, and a control sample comprised of pooled cotyledon tissue from seeds harvested in 2014. All assessments of RNA quality, including RIN calculations (described below), were consistent among control samples and confirmed there were no batch effects (data not shown).

### RNA characterization

The concentration of extracted RNA was measured by absorbance using a Nanodrop (Thermo Fisher, Wilmington, DE, USA) 1000 spectrophotometer. Samples were diluted to 1 ng µl^–1^ in water. Fragment sizes of the diluted RNA were assessed by electrophoresis using Agilent (Waldbronn, Germany) RNA 6000 Pico chips and the Plant RNA Pico assay (software version B.02.08.SI648 R3) according to the manufacturer’s protocols. Agilent 2100 software was used to draw electropherogram baselines and assess peak areas of different fragment sizes.

RNA integrity is initially estimated by the prevalence and ratio of the 25S and 18S rRNA fractions, which are the most abundant RNAs in a total RNA preparation. The ratio of 25S rRNA to 18S rRNA is 2:1 in high-quality RNA, while other prevalent RNAs are detected as discrete bands including plastid rRNAs, 5S rRNA, and tRNAs. Peak areas were normalized for each electropherogram to reflect the percentage of total RNA area encompassed by each peak. The effect of storage time on distribution of RNA fragment size was tested with ANOVA using JMP 12.2 (SAS Institute Inc., Cary, NC, USA).

Degradation of RNA is recognized as smears or additional peaks in electropherograms, indicating that molecules were fragmented. The RIN (Agilent Bioanalyzer software) considers the rRNA ratio, the height of the 25S rRNA peak, and the presence of peaks in interpeak regions (between the 25S and 18S rRNA peaks and between the 5S and 18S rRNA peaks) (Schroeder *et al.*, 2006). The plant-specific RIN calculation also accounts for the presence of plastid rRNA peaks in total RNA samples. The tolerance for 5S region anomalies was set to 1.0 for RIN calculations in MMY-extracted samples to account for high abundance of low molecular weight RNA.

### Statistical analyses

Student’s *t*-test was used to compare the mean RIN of embryonic axis and cotyledon RNA extractions from seeds harvested after 1999 (total germination >90%) and before 1999 (total germination generally <90%). We used ANOVA to test the effect of tissue type, age cohort, and germination percentage on RIN values, and correlated RIN values obtained for embryonic axes and cotyledons of the same seed with the total and apparently healthy germination percentage of a population of 60 seeds. To test whether RNA quality was informative regarding the germination potential of the seed, RNA was extracted from a small portion of the cotyledon cut from the seed before it was sown. Sown seeds (with a portion of the cotyledon removed) were then scored as germinating or not germinating, and RIN values from each class and cohort were compared using Student’s *t*-test. These statistical comparisons were performed in JMP 12.2.

## Results

### Germination physiology of stored seeds

**Fig. 1.**
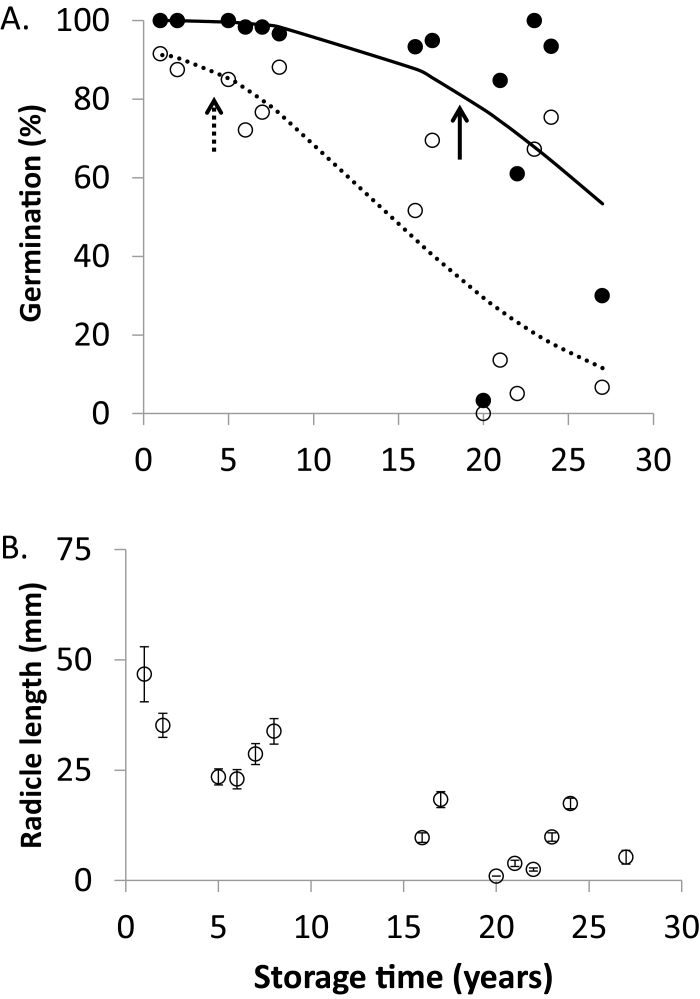
Seed quality of soybean (*Glycine max* cv. ‘Williams 82’) stored for up to 27 years at 5 °C and 35% RH. (A) Percentage germination. Filled circles show total germination, defined as radicle emergence through the seed coat; *n*=60 for each year. Open circles show apparently healthy germination, defined as a seedling with radicle >5 mm long. Curves were calculated using the Avrami function fitted to germination data (Walters *et al.*, 2005): the solid line shows the curve fitted to total germination, the solid arrow indicates the P80 for total germination (18.6 ± 0.6 years), the dashed line shows the curve fitted to apparently healthy germination, and the dashed arrow indicates the P80 for apparently healthy germination (4.5 ± 0.9 years). (B) Average radicle length of germinating seeds; only seeds with measurable radicles were included (*n* ranged from 2 to 60). Error bars show the SE of *n* germinating seeds.

Dry seeds from all age cohorts appeared the same, with no detectable difference in shape or size and no apparent browning in older seeds. Germination tests were required to detect differences among age cohorts. These assays revealed no differences among cohorts when tested within 6 months of harvest; initially, all seed lots gave total, apparently healthy, and radicle length measurements similar to values reported here for the 2015 cohort (1-year-old sample in Fig. 1; initial data for other harvest years not given). However, when retested in 2016, germination was still high for some seed lots and lower for others. In fact, the period between 17 and 27 years appeared to mark a transition in response to storage time from little detected change to rapid loss of germination potential. The logistic curve fit to the data (Mira *et al.*, 2016) indicated that germination declined to 80% (P80) in 18.1 ± 0.6 years (P50=24.6 ± 0.6 years; P10=34.9 ± 1.4 years) for this storage environment and cultivar of soybean. Consistent with the calculated P80, a comparison of seeds harvested after 1999 (within 17 years of storage) and before 1999 (after 17 years of storage) showed significantly different germination proportions [Fig. 1A, filled circles; *P*<0.05, tested with analysis of means for variances–Levene (absolute deviation from the median) to account for a non-normal distribution; Brown and Forsythe (1974)]. In contrast, the deterioration time course for apparently healthy germination was more linear, giving the impression that an aging response could be observed within a few years of storage based on increasing incidence of stunted seedlings (Fig. 1A, open circles). This is reflected in the reduced P80 of 4.8 ± 0.9 years (P50=15.2 ± 0.6 years; P10=31.8 ± 1.5 years), as well as the quick decline in radicle growth with age (Fig. 1B). However, the number of germinated seeds required and the strong influence of germination conditions (not shown) reduce the sensitivity of the radicle assay to subtle changes in seed health. It was not possible to predict seed germination behavior (i.e. those that did not germinate, germinated slowly or grew vigorously) from dry or imbibing seeds.

### RNA characteristics of dry soybean seeds

RNA yield varied considerably among tissues (Table 1). RNA yield from seed coats (average of 7 ± 1.7 ng mg^–1^ tissue among age cohorts) was much lower than RNA yield from plumules (average of 6377 ± 1184 ng mg^–1^ tissue among age cohorts). RNA yield from embryonic axes (including plumule) was greater than that from cotyledons, which was much greater than that from seed coats. Based on the RNA yield per tissue, tissue contribution to the RNA signal from a whole soybean seed would be 72–87, 6–19, 13–28, and 0.2–0.3% for the cotyledon, plumule, embryonic axis, and seed coat, respectively. Though RNA yield tends to be lower in older (>17 years, harvested before 1999) compared with younger (≤17 years, harvested after 1999) cohorts, the differences in yield were not significant for any tissue (*P*>0.05 for each tissue, Student’s *t*-test).

**Table 1.** Characteristics of RNA extracted from different tissues of soybean seeds harvested after 1999 (germination >90%) and before 1999 (germination <90%), ±SE The two time ranges compare seed cohorts showing no effects of aging (‘after 1999’) and evidence that aging occurred (‘before 1999’)

Tissue	Mass of tissue per seed (mg)	RNA yield (ng per seed)		RIN			Number of samples	
		After 1999	Before 1999	After 1999	Before 1999	*P*-value	After 1999	Before 1999
Seed coat	15 ± 0.9	118 ± 51	80 ± 22	7.7 ± 0.4	3.7 ± 0.6	0.0004	6	4
Plumule	0.8 ± 0.1	7595 ± 2053	3532 ± 1208	6.4 ± 0.2	5.5 ± 0.2	0.0067	10	10
Embryonic axis	5.4 ± 0.2	3865 ± 889	3010 ± 590	7.7 ± 0.2	6.4 ± 0.2	0.0004	23	23
Cotyledon	186 ± 2.4	4898 ± 969	6692 ± 2062	7.9 ± 0.1	6.0 ± 0.3	<0.0001	23	25

**Fig. 2.**
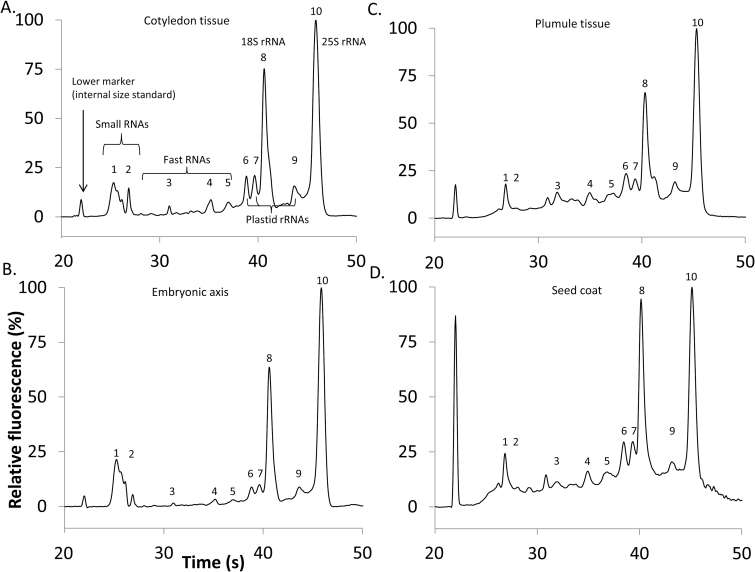
Representative electropherograms from dry tissues of soybean seeds harvested in 2014 showing exemplary RNA quality, transformed to show the percentage of maximum fluorescence for that sample. (A) Cotyledon tissue, extracted with the modified MMY method; RIN=8.1. (B) Embryonic axis, extracted with the modified MMY method; RIN=9.2. (C) Plumule tissue, extracted with the Qiagen RNeasy kit; RIN=7.3. (D) Seed coat, extracted with the Qiagen RNeasy kit; RIN=6.9.

Electrophoretic patterns from 2-year-old seeds (2014 harvest) reflected general characteristics of high-quality RNA extracted from plants (Fig. 2A, cotyledons). RNA fragment sizes ranged from 120 bp to 3045 bp, and the 18S (1730 bp, peak #8) and 25S (3045 bp, peak #9) rRNA peaks were prominent. Other identifiable fragments were plastid rRNA peaks (1400, 1500, and 2450 bp, peaks #6, 7, and 9) (Babu and Gassmann, 2016). Peaks #1 and 2 represent the small RNAs such as 5S rRNA and tRNA (Schroeder *et al.*, 2006). The remaining peaks (#3, 4, and 5) occur within the ‘fast’ area between the small RNAs and 18S rRNA. They cannot be assigned a definitive identity based on their size or shape. Electropherograms from all tissues sampled contained these same 10 distinct peaks (Fig. 2). For recently harvested seeds (1 and 2 years old; 2015 and 2014 cohorts), calculated RIN values of RNA extracted from seed coats, embryonic axes, and cotyledons were similar, ranging from 6.9 to 9.2. RNA extracted from plumules tended to give lower RIN values (ranging from 5.6 to 7.3) than RNA extracted from whole embryonic axes.

**Fig. 3.**
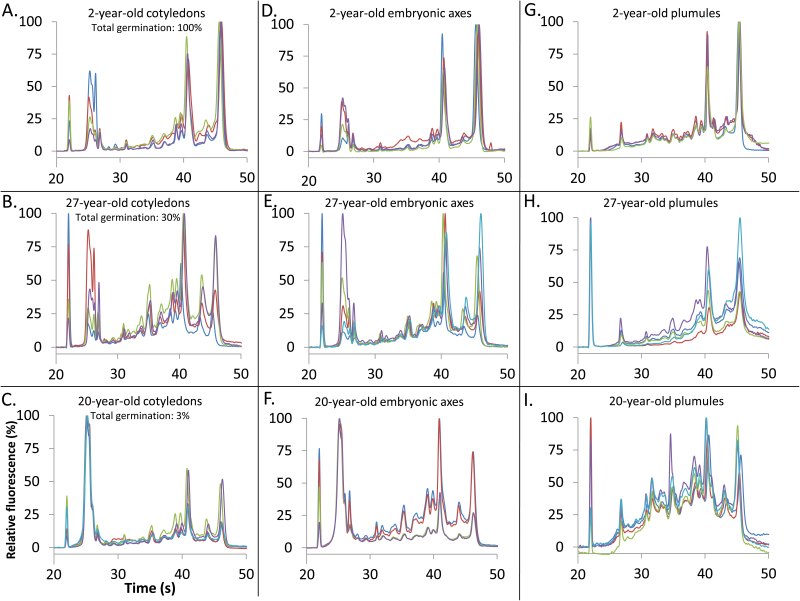
Electropherograms of RNA extracted from soybean seeds harvested in different years with different total germination percentages, transformed to show the percentage of the maximum fluorescence for that RNA sample. Each line indicates the results of a separate RNA extraction. (A) Two-year-old cotyledons (2014 harvest; *n*=4, average RIN=7.7); (B) 27-year-old cotyledons (1989 harvest; *n*=5, average RIN=5.5); (C) 20-year-old cotyledons (1996 harvest; *n*=5, average RIN=4.5); (D) 2-year-old embryonic axes (2014 harvest; *n*=4, average RIN=8.4); (E) 27-year-old embryonic axes (1989 harvest; *n*=5, average RIN=6.5); (F) 20-year-old embryonic axes (1996 harvest; *n*=4, average RIN=4.9); (G) 2-year-old plumules (2014 harvest; *n*=4, average RIN=6.6); (H) 27-year-old plumules (1989 harvest; *n*=5, average RIN=6.1); (I) 20-year-old plumules (1996 harvest; *n*=5, average RIN=4.9). RNA from cotyledons and embryonic axes was extracted using the MMY method, which allows detection of small RNAs that are not as prominent in extractions from plumules using the Qiagen RNeasy kit. Electropherograms from the 2014 cohort include data shown in Fig. 2. (This figure is available in colour at *JXB* online.)

**Fig. 4.**
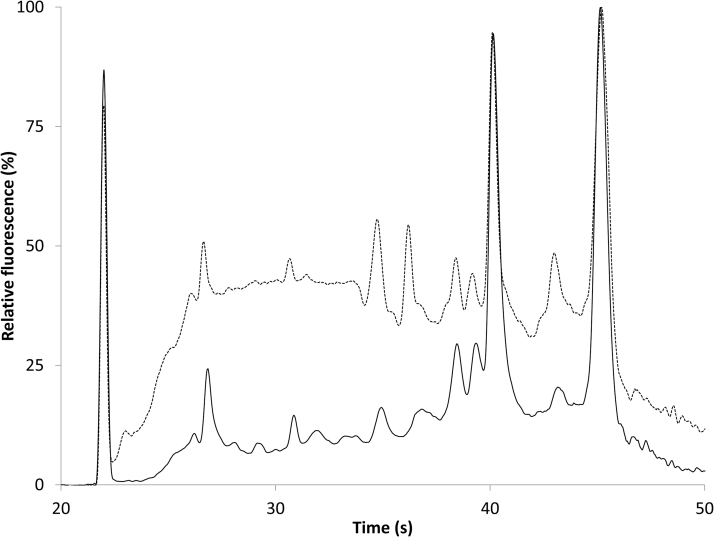
Electropherograms from seed coats, transformed to show the percentage of the maximum fluorescence for that RNA sample. Solid line, seed coat from 2-year-old seed (2014 harvest, RIN=6.9; also shown in Fig. 2D); dashed line, seed coat from 27-year-old seed (1989 harvest, RIN=4.7). Seed coats were extracted with the Qiagen RNeasy kit.

Broad differences in electrophoretic patterns were revealed by comparing RNA extracted from 2-year-old seeds (2014 cohort) with RNA extracted from 27- (1989 cohort) and 20- (1996 cohort) year-old seeds, the latter two cohorts having low total germination rates of 30% and 3%, respectively (Fig. 1). In general, RNA degradation is marked by a shift towards smaller fragments (Figs 3, 4, representative electropherograms); the size of the 25S rRNA peak (#10) was smaller in the 27- and 20-year-old samples compared with the 2-year-old samples, and the RNA signals in the fast region and the region between the 18S and 25S rRNA peaks were concomitantly larger. The fast RNA signal was weaker and the small RNA signal stronger in the nearly-dead 20-year-old sample (1996 cohort) compared with the 27-year-old sample with 30% germination (1989 cohort). RIN values of RNA extracted from cotyledons were 7.8 ± 0.2 (2014 cohort, *n*=4, variation=SE) (Fig. 3A–C), 5.5 ± 0.7 (1989 cohort, 30% viable seeds, *n*=5), and 4.5 ± 0.6 (1996 cohort, 3% viable seeds, *n*=5). RIN values of RNA extracted from embryonic axes were 8.4 ± 0.3 (2014 cohort, *n*=4), 6.5 ± 0.3 (1989 cohort, 30% viable seeds, *n*=5), and 4.9 ± 0.5 (1996 cohort, 3% viable seeds, *n*=4) (Fig. 3D–F). Electrophoretic patterns of RNA in plumules and seed coats from substantially aged seeds differed from those in the cotyledons and axes, in part as a result of the lower integrity and higher variability perceived in recently harvested seeds (Figs 3G–I, 4). Noticeable changes were observed in older seeds in the prominence of the 25S rRNA peak, the abundance of smaller RNAs, and the baseline shape. RIN values of RNA extracted from plumules (*n*=5 for each cohort) were 6.6 ± 0.4 (2014 cohort), 6.1 ± 0.1 (1989 cohort, 30% viable seeds), and 4.9 ± 0.1 (1996 cohort, 3% viable seeds). RIN values of RNA extracted from seed coats (*n*=2 for each cohort) were 6.9 (2014 cohort), 4.7 (1989 cohort, 30% viable seeds), and 2.85 (1996 cohort, 3% viable seeds). A summary of the mean RIN values for each tissue type pooled for seeds stored for ≤17 years (post-1999 harvest) and >17 years (pre-1999 harvest) demonstrates extensive change for seed coats (*P*<0.05, ANOVA), comparable change in RIN for cotyledons, embryonic axes, and plumules of seeds harvested post-1999 (*P*>0.05, ANOVA), and a significant effect of tissue type on RIN values for seeds harvested pre-1999 (*P*<0.0001, ANOVA) (Table 1).

**Fig. 5.**
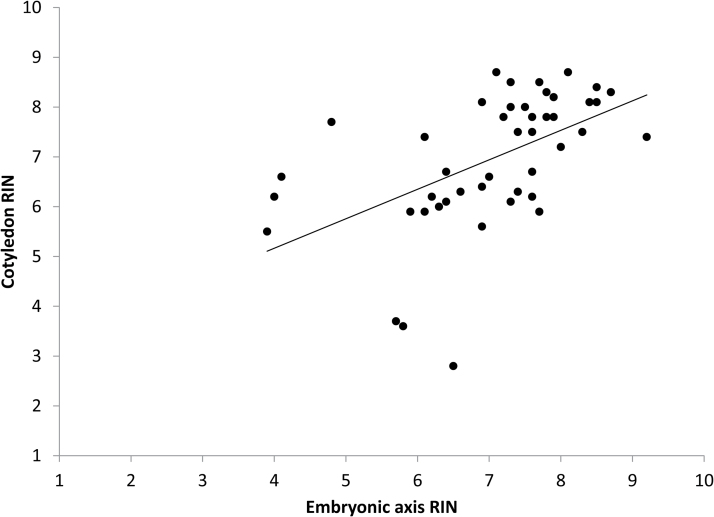
Correspondence of RIN values measured for cotyledons and embryonic axes of soybean seeds stored for up to 27 years. Each data point (*x*,*y* pair) represents an individual seed from one of 10 age cohorts harvested between 1989 and 2015. The correlation is positive: cotRIN=2.798 + 0.592×embryo RIN (*n*=48, df=47, *R*^2^=0.28, *F*=11.4, *P*=0.002).

To obtain a more detailed view of RNA quality, we selected 10 of the 14 age cohorts (four or five seeds from each cohort) to represent seed lots with total germination ranging from 100% to 3% (Fig. 1, seeds stored for 1, 2, 5, 8, 16, 20, 21, 22, 23, and 27 years). RNA was extracted from cotyledons and embryonic axes of the same seed, and RIN values ranged from 2.8 to 8.7 and from 3.9 to 9.2, respectively (Fig. 5). RIN values from the two tissues are correlated (*R*^2^=0.28, *n*=48, *P*=0.002, RIN_cot_=2.798 + 0.592×RIN_axis_), but there was considerable unexplained variation, reflected by the low *R*^2^ of the regression, non-zero intercept, and slope <1 (*P*<0.003 for intercept ≠0; *P*<0.0001 for slope ≠1).

**Fig. 6.**
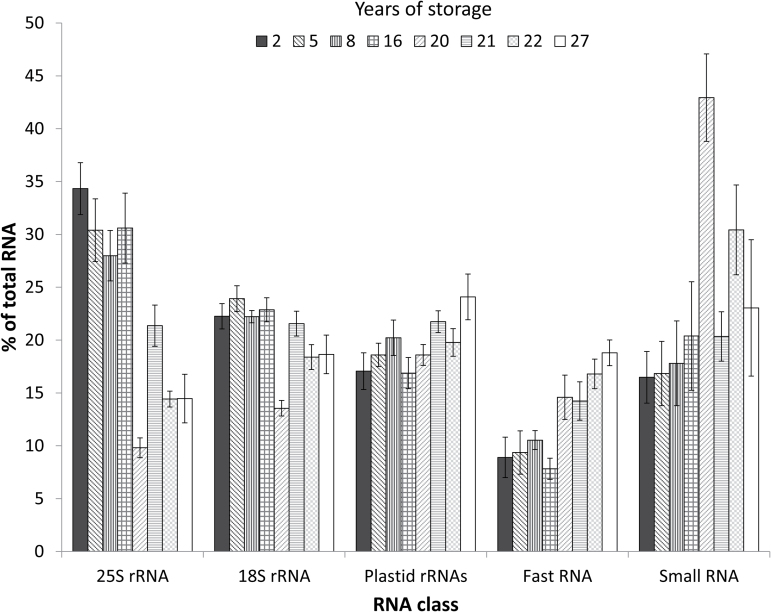
Seed aging effects on the size of RNA fragments. Each bar represents the percentage of the peak area within an RNA class compared with the total peak area for soybean seeds from eight different age cohorts (2014, 2 years of storage; 2011, 5 years of storage; 2008, 8 years of storage; 2000, 16 years of storage; 1996, 20 years of storage; 1995, 21 years of storage; 1994, 22 years of storage; 1989, 27 years of storage). Error bars represent the SE within age cohorts; the number of RNA extractions ranged from seven to nine per cohort (number of seeds=4 or 5).

The trend of decreasing large RNAs and increasing small RNAs with storage time was further characterized by calculating average peak areas for each RNA class (small, fast, plastid rRNA, 18S rRNA, and 25S rRNA) from the axis and cotyledon electropherograms for eight age cohorts (Fig. 6). As seed age increases, more RNA is found in the small and fast RNA classes, and less is found in the 25S rRNA and 18S rRNA classes (*F*=20.2, *P*<0.0001, ANOVA). The changes in the fast RNA and 18S rRNA classes are less pronounced than the changes in the small RNA and 25S rRNA classes. No change is detected in the relative abundance of plastid rRNAs.

### Correlation of changes in RIN and germination with storage time

**Fig. 7.**
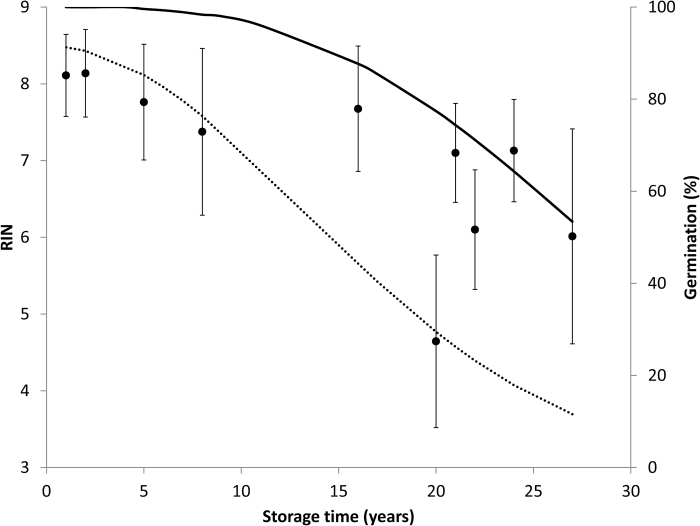
RNA quality of soybean (*Glycine max* cv. ‘Williams 82’) stored for up to 27 years at 5 °C and 35% RH. RNA quality is expressed as the mean RIN for 8–10 extractions from the embryonic axis and cotyledon of four or five seeds from different age cohorts (filled circles; error bars are the SE). Time courses for total (solid line) and apparently healthy (dashed line) germination are presented as the Avrami curves provided in Fig. 1A.

**Fig. 8.**
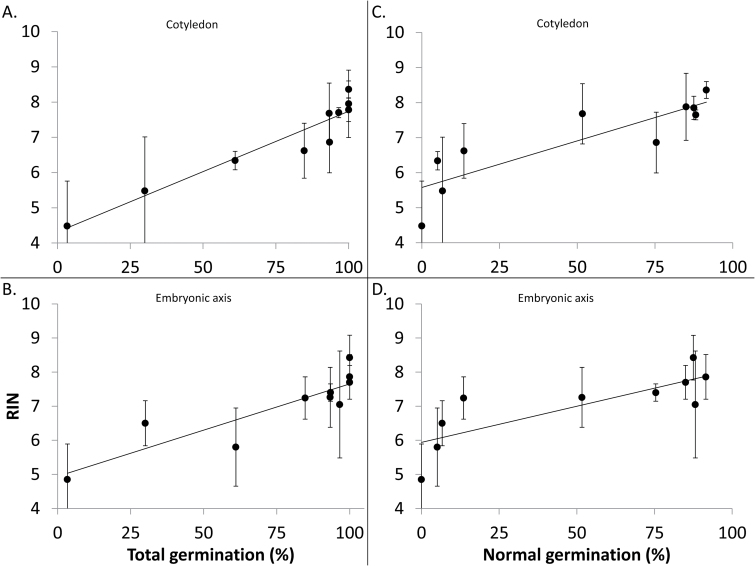
Correlation of RNA quality and germination behavior of soybean seeds stored for up to 27 years at 5 °C. RIN values are taken from Fig. 6 and germination data are from Fig. 1A. Error bars show the SD; *n*=10 for each correlation. (A) Total germination percentage and average cotyledon RIN: df=9, *R*^2^=0.91, *F*=79.4, *P*<0.0001; (B) total germination percentage and average embryonic axis RIN: *R*^2^=0.78, *F*=28.2, *P*=0.0007; (C) apparently healthy germination percentage and average cotyledon RIN: *R*^2^=0.74, *F*=23.18, *P*=0.0013; (D) apparently healthy germination percentage and average embryonic axis RIN: *R*^2^=0.66, *F*=15.2, *P*=0.0045.

Data presented herein show a clear decline in germination potential in soybean seeds over a 27 year period that became noticeable only after ~17 years of storage. The data also show the tendency for RNA fragments to decrease in size over the 27 year period, but the time course for RNA changes remains unclear: are changes noticeable before 17 years? There is no significant difference in mean RIN for age cohorts between 1 and 16 years [Fig. 7, *P*>0.05, analysis of means for variances–Levene (absolute deviation from the median)]. Changes in RNA integrity, as measured by RIN, only appear after year 16 (the mean RIN for samples harvested post-1999 is significantly different from the mean RIN for samples harvested pre-1999, Student’s *t*-test, *P*<0.0001). In fact, mean RIN values closely associate with total or apparently healthy germination percentage, though correlations were strongest for the linear regression between total germination and RIN from cotyledon extracts (Fig. 8A; *R*^2^=0.91, *n*=10, *F*=79.4, *P*<0.0001) and weakest for the linear regression between apparently healthy germination and RIN from axis extracts (Fig. 8D; *R*^2^=0.66, *n*=10, *F*=15.2, *P*=0.0045). Log-transforming germination data produced better correlations with apparently healthy germination (not shown).

### Comparisons of RIN in germinable and non-germinable seeds

**Fig. 9.**
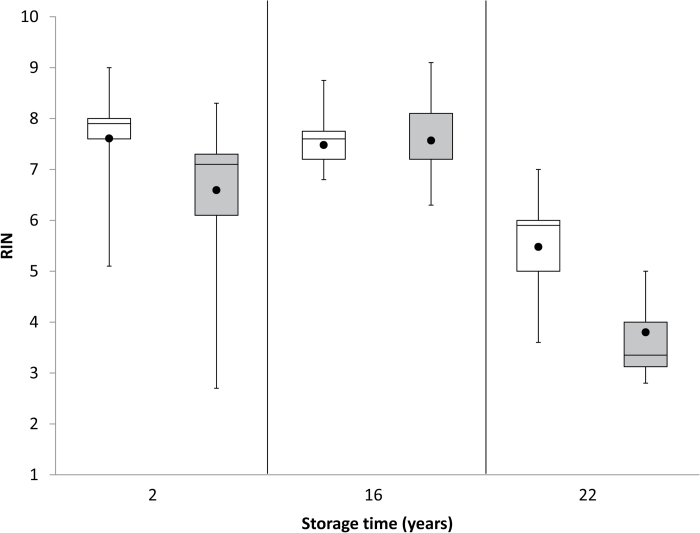
Box plots of RIN values of RNA extracted from soybean seeds that germinated (white boxes) and did not germinate (gray boxes) from three age cohorts: 2 years (2014, *n*=13 for alive; *n*=13 for dead); 16 years (2000, *n*=19 for alive; *n*=13 for dead), and 22 years (1994, *n*=9 for alive; *n*=8 for dead). RNA was extracted from a portion of the cotyledon clipped from seeds prior to the germination assay. Filled circles represent the mean RIN for each viability class and age cohort, the horizontal lines dividing each box represent the median RIN, and the vertical lines represent the range of RIN values measured for each treatment. At least 800 seeds were germinated in the 2 year age cohort to identify the few seeds that were not germinable.

The physical appearance of the dry seed does not help distinguish between seeds which are dead or alive, and so the transition between germinable and non-germinable seeds (in this case, ~17 years of storage) goes mostly unnoticed unless seeds are repeatedly viability tested or, as in this study, a unique collection of age cohorts is available for contemporaneous sampling. Our work shows that RIN in cotyledon tissues correlates closely with total germination (Fig. 8A, C). Therefore, we tested whether low RIN in the cotyledon could indicate seed mortality by extracting RNA from dry cotyledon pieces excised from seeds that subsequently germinated or failed to germinate. Seeds that did and did not germinate (Fig. 9, white and gray boxes, respectively) followed the same time course for RIN values as reflected in Fig. 7, and RIN values from samples which were dead or alive overlapped. In 22-year-old samples (1994 cohort), the mean RIN for germinable seeds (5.5) was significantly higher than the mean RIN for non-germinable seeds (3.8; *P*=0.008, Student’s *t*-test).

## Discussion

The results of this study illustrate why predictions of seed longevity and early detection of seed aging are elusive. Under fairly routine storage conditions, the transition from asymptomatic to rapid aging took ~5 (stunting) to 17 (lost viability) years in a seed known to be short lived (Fig. 1). However, because of high variation among seed lots, this transition cannot be recognized until the complete deterioration time course is available to see overall trends. Changes in RNA integrity, measured by RIN, track the changes in seed germination potential closely (Figs 7, 8) and, arguably, represent the tightest published correlation between the definitive symptom of seed aging (i.e. lost germination potential) and changes in a chemical constituent (Fu *et al.*, 2015). Our results build from earlier studies indicating relationships between RNA integrity and seed viability (Bray and Chow, 1976; Brocklehurst and Fraser, 1980; Kranner *et al.*, 2011; Chen *et al.*, 2013). However, our work provides novelty by using new quantification methods to evaluate RNA degradation, as well as dry-stored seeds, which are more representative than humid-stored seeds of the actual aging process under investigation (Rajjou *et al.*, 2008; Schwember and Bradford, 2010; Ballesteros and Walters, 2011; Agacka-Mołdoch *et al.*, 2016). Humid-stored seeds are commonly used to circumvent the inconvenience of long waiting times to observe symptoms; our study was possible because of access to seed inventories harvested over multiple years.

RNA integrity was measured by the proportion of sizes of RNA fragments within an extract. In principle, this is similar to well-studied effects of time on DNA quality, in which progressive fragmentation is anticipated over a much longer time period (Walters *et al.*, 2006; Rizzi *et al.*, 2012). Faster DNA breakdown is a measure of pathology and is diagnosable using a number of assays including the ‘comet’ assay, named for the appearance of high concentrations of large molecular weight DNA followed by a smear of low molecular weight DNA fragments (Neumann *et al.*, 2009; Lavelle and Foray, 2014). Analogously, RNA degradation is diagnosed by the replacement of distinct large molecular weight RNAs with a smear of low molecular weight RNA fragments (Schroeder *et al.*, 2006; Gallego Romero *et al.*, 2014; Malentacchi *et al.*, 2014). Because the size distribution of intact RNA molecules is more diverse than DNA, distinguishing fragmented large RNAs from intact, smaller mRNAs can be challenging and may require a combination of methods. The tendency for RNA to fragment during dry storage is unlikely to be through enzyme catalysis. We speculate that these fissures arise through free radical-mediated oxidation, as has been suggested for other cellular constituents (Walters *et al.*, 2010). The tight correlation between percentage germination and average RIN (Fig. 8) suggests that RNA degradation and seed aging occur at similar rates; however, the lack of correspondence between RIN values for seeds which were dead or alive seeds (Fig. 9) is a confirmation to us that the switch between ability and inability to germinate is not driven by RNA integrity.

A striking feature of the germination time course is the variation in germination behavior among seed lots (age cohorts) once the viability threshold (near 17 years) is surpassed. For example, the germination percentage of the 20 year cohort is near 0 and germination percentages of the 23 and 24 year cohorts are similar to those of recently harvested seeds (Fig. 1A). RNA integrity appears to track this variation, with noticeably lower RIN in the 20 year cohort compared with older seeds having both higher germination and higher mean RIN (Fig. 7). Causes of variation in longevity among seed lots—and, indeed among individual seeds—are not well understood, but have been attributed to genetic and maternal effects, and pre- and post-harvest treatments prior to storage (Schwember and Bradford, 2010; Rajjou *et al.*, 2012; Agacka-Mołdoch *et al.*, 2016). These factors contribute to the difficulty of phenotyping the longevity trait and to the requirement for viability monitoring in seedbanks (FAO, 2014; Fu *et al.*, 2015). The few individuals within an aged seed lot that have high germination and high RIN may also have more effective mechanisms of protection against aging than their dead counterparts, though we cannot rule out random effects arising from idiosyncrasies of the assay or error associated with sampling a small portion of the seed. RNA integrity measurements are fairly easy to control and standardize, and may offer a less subjective way to monitor the health of seeds stored over decades. Alternative assays involving growth measurements such as radicle length (Fig. 1B) are highly affected by seed traits (seed coat thickness, embryo maturity, dormancy) and germination conditions, which reduces the sensitivity to overall seed health.

Our conclusion about the close correlation of RIN with germination potential among age cohorts is based on RNA analyses of embryonic axes and cotyledons (Figs 3–8). Higher variation in RNA integrity was noted in plumules, and pronounced change with time was noted in seed coats (Table 1), suggesting that these tissues could show quite subtle effects of time. In soybean, embryonic axes and cotyledons appeared to have similar initial RIN values and similar kinetics of RNA degradation (Figs 2, 3, 5). This presents a current opportunity to correlate RIN of cotyledon tissue with physiology of the associated embryonic axis from an individual seed (Fig. 9), as well as future opportunities to use RNA analyses in non-destructive assays to predict seed performance.

The question of a ‘death signature’ is prominent for cryptobiotic organisms such as dry seeds (Neumann *et al.*, 2009; Segev *et al.*, 2012; Fu *et al.*, 2015). RIN values of <3.5 probably reflect mortality in soybean seeds (Fig. 9). It remains to be seen if death is marked by a similar RIN value in other cultivars or species. Finding indications of mortality using a chemical assay is a pronounced advance because death could only be truly diagnosed by the eventual lack of physical integrity of tissues in imbibed, non-germinating seeds (i.e. dead seeds eventually become mushy) (Baskin and Baskin, 2014). In wild populations, slow germination or seed dormancy are common, and this physiology is often indistinguishable from dead seeds in a germination test (Baskin and Baskin, 2014). Therefore, RNA integrity may provide useful insights.

Detection of dying seeds is another profound issue. There is very little distinction in RIN among seeds which are alive or dead within healthy age cohorts (Fig. 9), leading us to suggest that although seeds with degraded RNA are dying, failure to germinate normally can have causes unrelated to RNA integrity. In other words, time affects germination potential and RNA integrity in similar but not identical ways; the two measurements are responses that co-correlate with time, but there is no indication yet of a causal relationship between RNA degradation and lost viability. Future studies will use transcriptome profiling as a more fine-grained analysis to determine if loss of germination-specific transcripts distinguishes seeds that germinate from those that fail to germinate, or unaged seeds from aged seeds (Rajjou *et al.*, 2012; Waterworth *et al.*, 2016). Alternatively, aging may be a syndrome of accumulated damage, and the ultimate event that triggers failure to germinate may be minor.

Our results also point out that germination assays only provide a snapshot of seed health and do not inform about the viability threshold that both defines seed longevity and clearly varies among age cohorts. Similarly, RNA integrity does not portend the upcoming aging threshold. The ultimate goal of finding a biological marker for longevity is more attainable now with a good marker of seed deterioration.

## Closing remarks

The relative sizes of RNA molecules decrease with storage time in soybean seeds stored under dry, refrigerated conditions for up to 27 years. This is an indication that RNA is degrading with time, presumably by oxidative reactions, and this degradation occurs contemporaneously with progressive loss of germination potential within the seed lot. The work provides some exciting possibilities to explore cryptobiotic organisms, in which changed physiological status is not observable until samples are thawed or water is added. The work also has some extremely practical implications in the context of phenotyping the seed longevity trait as well as ensuring that banked seeds can be monitored for health but that the collection is not depleted by that effort.

## Abbreviations:

PVP-40: polyvinylpyrrolidone-40
RIN: RNA integrity number.
